# Supplementary material for: Gating shift to an ohmic mode of TREK-1 channel induced by docosahexaenoic acid
Source: Biophys J. 2026 May 6;125(13):3286–98. doi: 10.1016/j.bpj.2026.05.005 (PMC13507441; doi:10.1016/j.bpj.2026.05.005)
Supplement: Supplementary file 1 [file mmc1.pdf]

Biophysical Journal, Volume *125*

Supplemental information

Gating shift to an ohmic mode of TREK-1 channel induced by docosa-hexaenoic acid

Emilie Bechard, Jean-Yves Le Guennec, and Marie Demion

SUPPLEMENTARY DATA

Supplementary Information 1: Th python script used to generate **Figure 7** is open-source (MIT license), hosted on GitHub, and available at the following link: https://github.com/EmilieDrim/2025_Model-of-TREK-1-activation-by-PUFAs

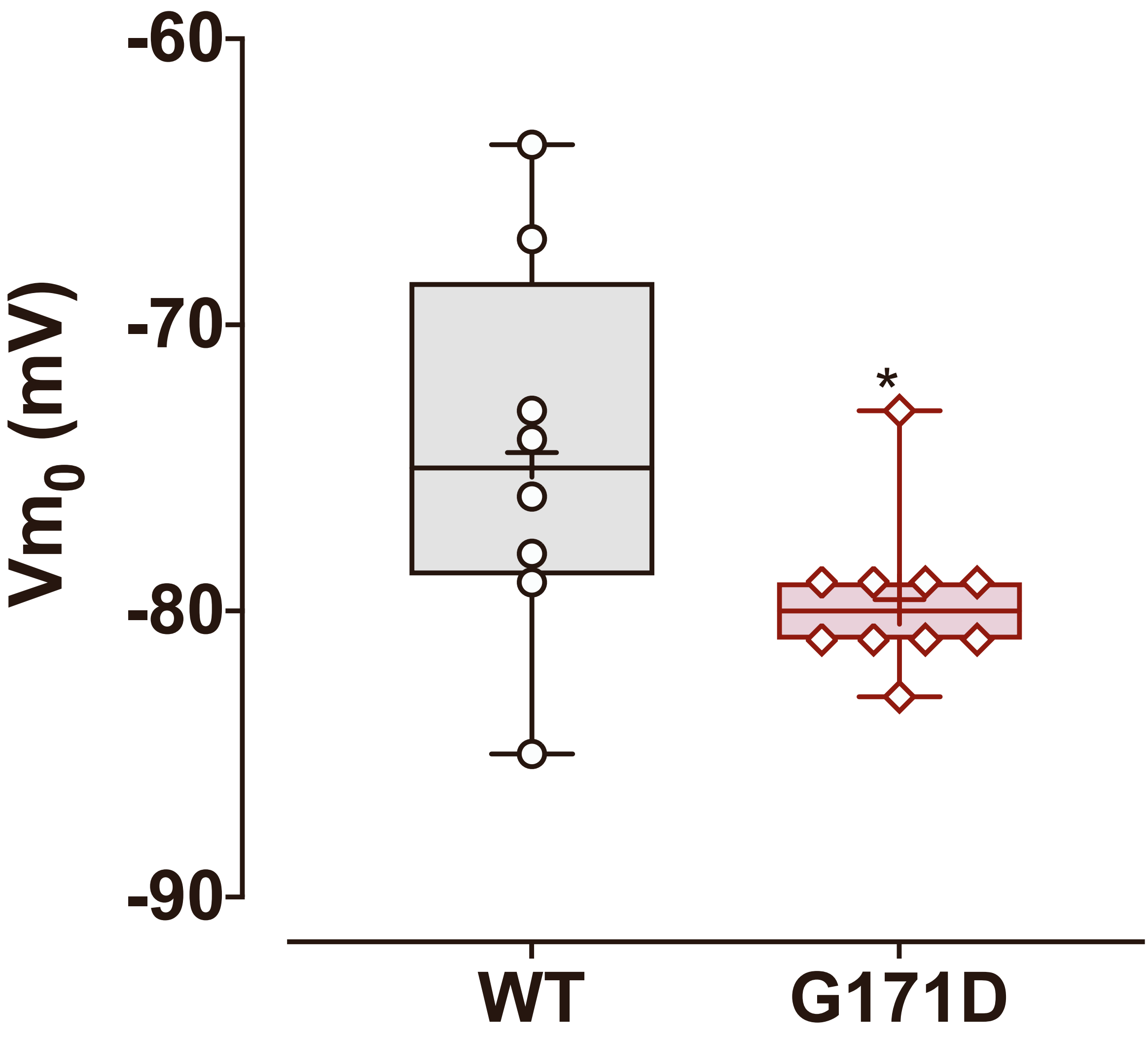

Supplemental data 1: G171D mutation hyperactivate TREK-1 channel
Resting membrane potentials of HEK293T cells transfected with WT TREK-1 (dark circles) or G171D TREK-1 (red diamonds) in resting conditions. Box plots display the mean (cross), median (horizontal line) and individual data points (circles).

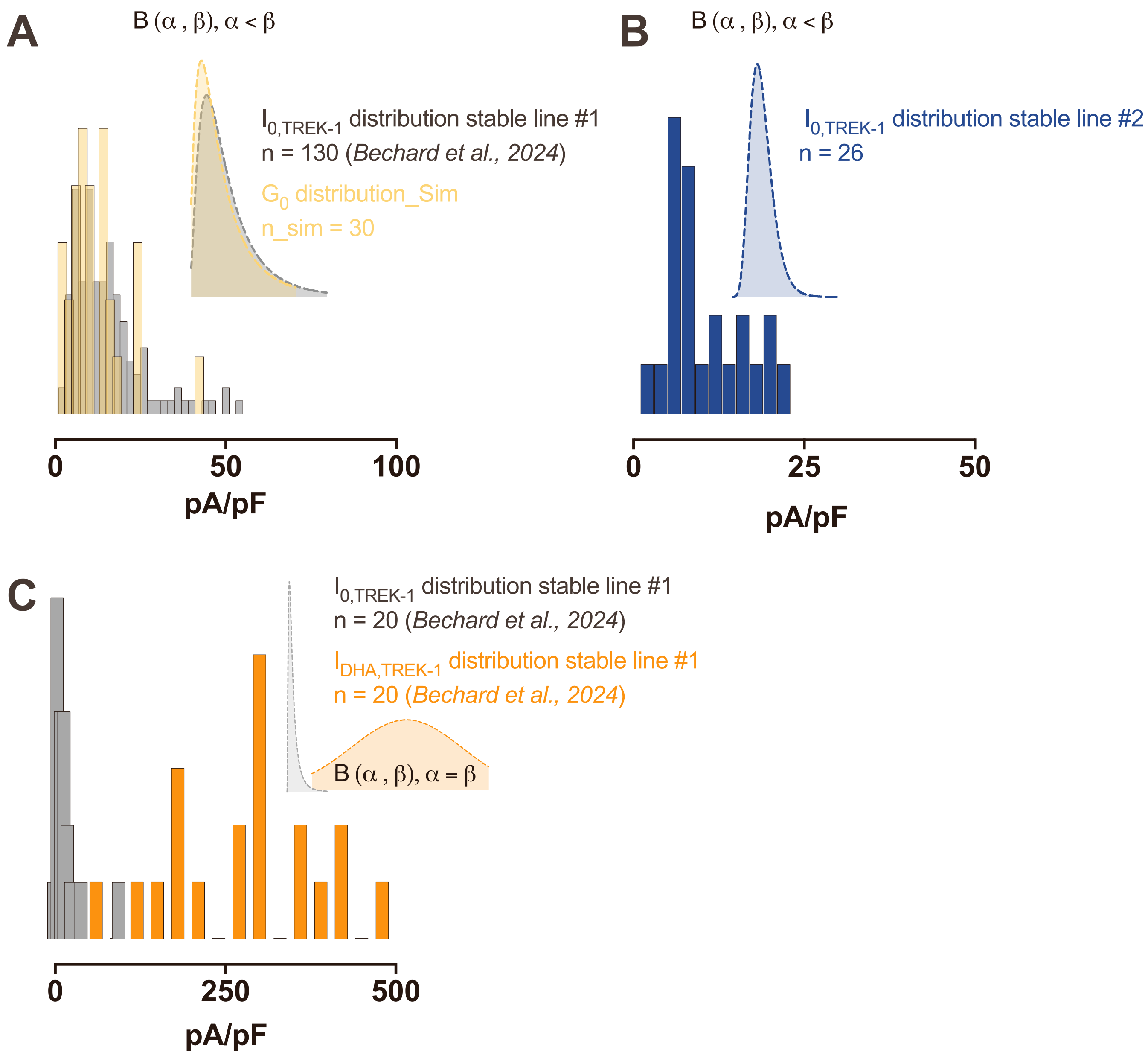

Supplemental data 2: Distributions of TREK-1 activity in HEK cell models

A. Distribution of TREK-1 current ($I_{0,TREK-1}$) in control conditions recorded at 0 mV from a stable HEK293 #1 line overexpressing TREK-1 channel (gray boxes, data from Bechard et al., 2024). Yellow boxes represent the distribution of modeled G_0 in Figure 7. **B.** Distribution of TREK-1 current ($I_{0,TREK-1}$) in control conditions recorded at 0 mV from a second HEK293 #2 stable line overexpressing TREK-1 channel (blue boxes). **C.** Superposition of $I_{0,TREK-1}$ (gray boxes) and $I_{DHA,TREK-1}$ (orange boxes) distributions, recorded from the HEK293 #1 stable line overexpressing TREK-1 (Bechard et al., 2024).

	Cellular model	Reference	$I_{max}/I_{0\text{-}WT}$	Linearity coefficient of $I_{0,WT}$	Linearity coefficient of $I_{DHA,WT}$
**DHA**	Transient transfection	Present study	16.58 ± 4.28	0.14 ± 0.01	0.67 ± 0.07
**DHA**	Stable HEK TREK-1 line	Bechard et al., 2024	29.79 ± 4.37	0.13 ± 0.009	0.58 ± 0.05
**LA**			24.76 ± 3.33	0.12 ± 0.008	0.53 ± 0.03
**ETA**			15.66 ± 18.78	0.12 ± 0.04	0.17 ± 0.07
**ML402**			9.56 ± 0.97	0.10 ± 0.01	0.21 ± 0.02

Supplemental Table 1: Linearization of TREK-1 current during activation by PUFAs and ML402.
