# Supplementary material for: Gating shift to an ohmic mode of TREK-1 channel induced by docosahexaenoic acid
Source: Biophys J. 2026 May 6;125(13):3286–98. doi: 10.1016/j.bpj.2026.05.005 (PMC13507441; doi:10.1016/j.bpj.2026.05.005)
Supplement: Supplementary file 2 [file mmc2.pdf]

Biophysical Journal
Article

Biophysical Society

Gating shift to an ohmic mode of TREK-1 channel induced by docosahexaenoic acid

Emilie Bechard,[1,*] Jean-Yves Le Guennec,[1] and Marie Demion[1]
[1]PhyMedExp, INSERM U1046, CNRS UMR9412, University of Montpellier, Montpellier, France

ABSTRACT The TREK-1 channel is a polymodulated K2P channel that responds to a wide range of physical and chemical stimuli, enabling it to contribute to the regulation of cellular excitability. This member of the TREK sub-family exhibits an outward rectification resulting from voltage-dependent gating, which can be reversibly converted to an ohmic leak mode by both synthetic and natural modulators. The present study aimed to investigate the gating mechanism in response to docosahexaenoic acid (DHA), using transient transfections of either the wild-type (WT) TREK-1 channel or its G171D variant at the transmembrane (TM)2.6 position in HEK 293T cells. We reported that DHA enhances the WT TREK-1 current by increasing the proportion of channels in a conductive state across the entire membrane potential (Vm) range, effectively shifting TREK-1 into an ohmic mode. Furthermore, we observed that disrupting the C-type gate function blunted DHA-induced activation, suggesting that DHA acts by stabilizing the selectivity filter that may involve TM2. Additionally, we observed considerable variability in the initial activity of TREK-1 under control conditions, which accounts for the heterogeneous effects of DHA among cells. These findings highlight the complex gating mechanism of TREK-1 and reveal a dynamic mechanism by which DHA, and likely other polyunsaturated fatty acids (PUFAs), modulate Vm and influence cellular excitability.

SIGNIFICANCE TREK-1 is a potassium channel that regulates cellular excitability in both the brain and the heart. It can be activated by polyunsaturated fatty acids such as docosahexaenoic acid (DHA), known for being neuro- and cardioprotective. We found that DHA activates the TREK-1 channel to varying extents, depending on the initial TREK-1 activity. Using a conductance model, we show that DHA can switch the TREK-1 channel from a voltage-dependent gating mode to a passive, ohmic "leaky" mode. This transition likely involves structural changes in the TM2 segment and in the C-type function of the TREK-1 protein, which are affected by the G171D mutation. Such variability in activation and gating behavior could critically influence the protective roles of the TREK-1 channel in physiological and pathological contexts.

INTRODUCTION

Potassium channels are classified into three main types: voltage-gated channels (Kv), inwardly rectifying channels (Kir), and two-pore-domain channels (K2P). Kv channels contribute to membrane repolarization of excitable cells, whereas background and/or leak channels primarily stabilize the resting membrane potential. The K2P family comprises 15 highly regulated channels widely expressed in excitable cells. These channels exhibit constitutive activity at rest, bringing the membrane potential close to the equilibrium potential of K^+ (E_K) and suppressing excitatory responses. K2P channels form dimers and share a common structure comprising four transmembrane segments (TM1–TM4), two pore-forming loops (P1 and P2), and intracellular N- and C-termini. Six sub-families are distinguished by sequence homology and the stimuli to which they respond. Often described as leak channels, their outward rectification is thought to arise from the asymmetric gradient of K^+, as predicted by the Goldman-Hodgkin-Katz equation.[1] However, almost all the K2P channels display voltage-dependent behavior, described as a flux-gated mode, in which channel activity increases at depolarized potentials according to the driving force. This ion-flux gating accounts for their outward rectification.[2,3] A reversible interconversion of TREK-1 between voltage-dependent mode and leak mode occurs depending on phosphorylation and dephosphorylation pathways.[2] The TREK sub-family channels (TREK-1/K2P2.1, TREK-2/K2P10.1, and TRAAK/K2P4.1) are expressed in excitable tissues,[4–8] contributing to several sensory transduction processes such as mechanosensitivity,[9,10]

Submitted August 18, 2025, and accepted for publication May 4, 2026.

*Correspondence: etu.emilie.bechard@gmail.com

Editor: Carlos Alberto Villalba-Galea.
https://doi.org/10.1016/j.bpj.2026.05.005

© 2026 The Authors. Published by Elsevier Inc. on behalf of Biophysical Society.
This is an open access article under the CC BY license (http://creativecommons.org/licenses/by/4.0/).

thermosensitivity,[11,12] nociception (Lengyel et al., 2020),[5,13] and analgesia.[4,14,15] The modulation of TREK-1 by a plethora of stimuli that alter its gating mode makes it an attractive therapeutic target for neuroprotection[16] and cardioprotection[17] (Bechard et al., 2022).[18] In this way, polyunsaturated fatty acids (PUFAs) are potent neuroprotective and cardioprotective agents by activating TREK-1 (Lu et al., 2017).[19,20] In a previous study comparing multiple PUFAs, we showed that PUFA-mediated activation of TREK-1 likely involves a direct interaction and that the omega-3 docosahexaenoic acid (DHA; C22:6Δ[2,10,12,21,22,23]) is among the strongest activators of TREK-1.[24] Unexpectedly, we also observed considerable variability in the magnitude of activation induced by the ten PUFAs tested. Here, we further investigated the opening mechanism of TREK-1 induced by PUFAs to determine whether this variable effect of PUFAs could influence TREK-1 function in cells.

MATERIALS AND METHODS

Cell culture

HEK293T cells were transfected with pIRES2 plasmids containing the coding sequences of wild-type (WT) hTREK-1 (*KCNK2*) or the G171D mTREK-1 variant (6 ng/μL). Transient transfections were performed using the JetPEI kit (Ozyme) according to the manufacturer's recommendations. Cells were seeded in 35 × 10 mm dishes (Falcon) 48 h before electrophysiological experiments and grown in an atmosphere of 95% air/5% CO_2. Dulbecco's modified Eagle's medium supplemented with GlutaMAX (Invitrogen, Cergy-Pontoise, France) was supplemented with 10% (v/v) heat-inactivated fetal bovine serum.

Electrophysiology experiments

TREK-1 currents (WT and G171D) were recorded in the whole-cell configuration of a patch clamp. Patch pipettes were filled with a solution containing 155 mM KCl, 3 mM $MgCl_2$, 5 mM EGTA, and 10 mM HEPES (pH adjusted to 7.2 using KOH). The extracellular solution contained 150 mM NaCl, 5 mM KCl, 3 mM $MgCl_2$, 1 mM $CaCl_2$, and 10 mM HEPES (pH adjusted to 7.4 using NaOH). Patch pipettes were pulled from borosilicate glass capillaries using a two-stage vertical puller (PC-10, Narishige), yielding pipette resistances between 2.4 and 4 MΩ. Experiments were performed at room temperature. Cells were continuously perfused during the time course of the experiments at a rate of 1–1.5 mL/min with extracellular solution alone or supplemented with 10 μM DHA. The bath volume was maintained between 2.5 and 3 mL using a vacuum system connected to a peristaltic pump (ISMATEC).

For current recordings, cells were clamped at a holding potential of −80 mV, followed by a 50 ms hyperpolarizing step to −100 mV and a depolarizing voltage ramp from −100 to +30 mV over 800 ms. The depolarizing voltage ramp was repeated every 10 s for 30 s prior to DHA perfusion and continued until a steady state was reached. Series resistance (Rs) was monitored throughout the experiment, and recordings were excluded from analysis if Rs exceeded 8 MΩ.

Currents were acquired using an Axopatch 200B amplifier (Axon Instruments), low-pass filtered at 5 kHz with an 8-pole Bessel filter, and digitized at 20 kHz using a Digidata 1550B (Axon Instruments). Data acquisition and stimulation protocols were controlled using pClamp10.7 software (Axon Instruments). In the results section, current magnitudes are expressed as current densities (pA/pF) measured at 0 mV. All whole-cell recordings were obtained from independent cells originating from different batches of transient transfection.

The linearity coefficient of the TREK-1 current-voltage relationship between −100 and +30 mV was calculated based on the ratio of slopes determined from the reversal potential (V_{rev}) as follows:

$$\mathrm{slope}_{-100\mathrm{mV}} = \frac{I_{-100\mathrm{mV}}}{-100 - V_{Rev}}, \tag{1}$$

$$\mathrm{slope}_{+30\mathrm{mV}} = \frac{I_{+30\mathrm{mV}}}{30 - V_{Rev}}, \text{ and} \tag{2}$$

$$\text{linearity coefficient} = \frac{\mathrm{Slope}_{-100\mathrm{mV}}}{\mathrm{Slope}_{+30\mathrm{mV}}}. \tag{3}$$

The linearity coefficient is defined within the interval [0;1] considering that the current does not flow toward an inward rectification. As a quality control, all linearity coefficients obtained from recordings showing a concomitant increase in Rs were excluded from the analysis.

Chemical

DHA (Sigma Aldrich, France, D2534) was dissolved in absolute ethanol to prepare a 10 mM stock solution and stored at −80°C. On the day of the experiment, DHA was diluted in extracellular solution to a final concentration of 10 μM.

Modeling of the channel activation process

Simulations of conductance dynamics (Figure 7) were performed using Python 3.11 with custom scripts developed in Jupyter Notebook (Text S1). Data generation and mathematical modeling were implemented using standard scientific libraries, including NumPy and SciPy for numerical computations and Matplotlib for data visualization.

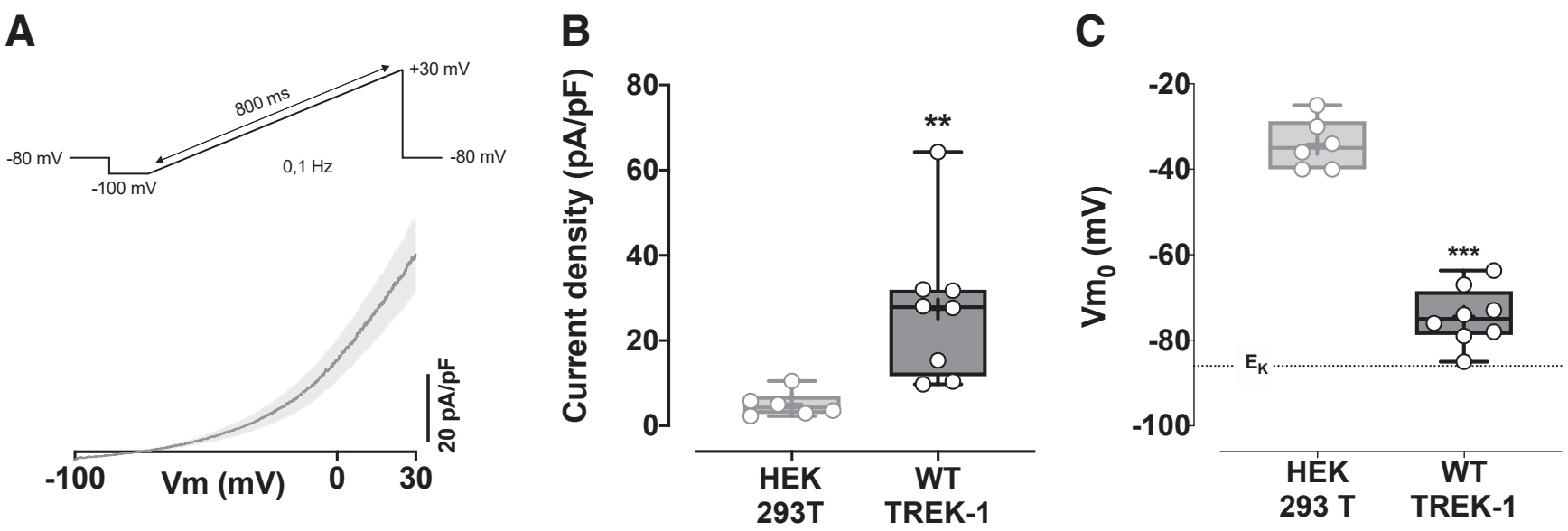

Figure 1. TREK-1 activity in HEK293T cells overexpressing the channel. (A) The top image depicts the voltage-ramp protocol used to elicit the TREK-1 current in the whole-cell configuration of the patch clamp in HEK293T cells transiently transfected with a plasmid encoding the TREK-1 channel (HEK-TREK-1_{WT}). Cells were clamped at a holding potential of -80 mV, followed by a 50 ms hyperpolarizing step to -100 mV, and then a depolarizing ramp to $+30$ mV over 800 ms. The protocol was repeated every 10 s (0.1 Hz). The bottom image shows the spontaneous IV curve of the initial TREK-1 current recorded in HEK-TREK-1_{WT} cells (mean $\pm$ SEM, $n = 8$). (B) Current densities measured at 0 mV in native HEK293T cells (*light gray*, $n = 6$) or in HEK-TREK-1_{WT} (*dark gray*, $n = 8$). (C) Resting membrane potential of untransfected HEK293T cells and HEK-TREK-1_{WT} cells under initial conditions. (B and C) Boxplots display the mean (*cross*), median (*horizontal line*), and individual data points (*circles*).

The kinetics of conductance activation (G) were modeled using the following sigmoidal function:

$$G_{(t)} = \text{bottom} + (\text{top} - \text{bottom}) \,.\, (1 + e^{-t/\tau})^n$$

Modeling of the ventricular action potential

To assess the effects of $I_{TREK\text{-}1}$ on the human ventricular action potential, a TREK-1 current was incorporated into the human ventricular action potential model developed by Ten Tusscher et al.[25]

The TREK-1 current was described by the following equation:

$$I_{TREK-1} = G_{TREK-1} \cdot \frac{1}{1 + e^{\frac{-(V_m - 86)}{\text{Linear}}}} \cdot (V_m - E_K),$$

where Linear was set to 200 mV to mimic a leaky current and to 50 mV to reproduce the outwardly rectifying $I_{TREK\text{-}1}$. $G_{TREK\text{-}1}$ was set to 0.055 nS/pF for the outwardly rectifying current and to 0.0314 nS/pF for the leaky current, such that the current magnitude was identical at $+30$ mV.[26] Under these conditions, changes in current magnitude occur between the potassium reversal potential and $+30$ mV (see Figure 8).

Statistical analysis

Statistical analyses were performed using GraphPad Prism 9 software. Nonparametric statistical tests appropriate for the data were applied: the Wilcoxon test for paired data and the Mann-Whitney test for unpaired data. A *p*-value ≤ 0.05 was considered statistically significant, with *p*-value significance indicated as follows: $^*p < 0.05$, $^{**}p < 0.01$, $^{***}p < 0.005$, and $^{****}p < 0.0001$. Correlations between two parameters were assessed using the Spearman correlation test, and the correlation coefficient r is reported in the results section when the *p*-value indicates significance (*p*-value $\leq$ 0.05). Linear regression was performed when the condition of the correlation was primarily verified by the Spearman correlation test. The *p*-value indicates the significance of the relationship, and the goodness of fit is given by the R^2 value. Error bars represent the 90% confidence intervals.

RESULTS

Initial variability in WT TREK-1 activity influences the intensity of PUFA-mediated activation

In this study, we used HEK293T cells transiently transfected with plasmids encoding either the WT TREK-1 channel (*KCNK2* gene, referred to as HEK-TREK-1_{WT}) or the G171D variant (HEK-TREK-1_{G171D}). TREK-1 currents were recorded in the whole-cell configuration of the patch-clamp technique using a voltage-ramp protocol from -100 to $+30$ mV (Figure 1A). Consistent with our previous observations in a HEK hTREK-1 stable cell line,[24] we observed a large variability in the initial activity of HEK-TREK-1_{WT} ($I_{0,WT}$). The average initial current at 0 mV ($I_{0,WT}$) was 27.4 $\pm$ 17.5 pA/pF (mean $\pm$ SD), ranging from 9.7 to 64.3 pA/pF in the initial condition (Figure 1B) and corresponding to a coefficient of variation (CV) of 63.9% ($n = 8$). The initial membrane potential in HEK-TREK-1_{WT} ($Vm_{0,WT}$) was -74.5 ± 6.6 mV, closer to the theoretical equilibrium potential of potassium ($E_K = -86.5$ mV) than in untransfected HEK293T cells ($Vm_{HEK293} = -34.2 \pm 5.9$ mV) (Figure 1C). Perfusion with 10 µM DHA (C22:6 $\Delta^{2,10,12,21,22,23}$) strongly increased the TREK-1 current from 27.4 $\pm$ 17.5 to 310.1 $\pm$ 83.2 pA/pF at 0 mV (Figure 2A).

TREK-1 activation by DHA produces a strong hyperpolarization, shifting the membrane potential ($Vm_{DHA,WT}$) toward -84.3 ± 1.8 mV (Figure 2B). The magnitude of the DHA effect is given by the ratio $I_{DHA,WT}/I_{0,WT}$. On average, this activation ratio was 16.6 $\pm$ 12.1, ranging from a 4.9- to 36.7-fold increase, with a CV of 73.1% (Figure 2C). As previously observed in the HEK hTREK-1 stable cell line,[24] variability in $I_{DHA,WT}/I_{0,WT}$ is partly explained by the variability in $I_{0,WT}$. The relationship between these two parameters is linearized using a log10 transformation (Figure 2C). Transforming both the

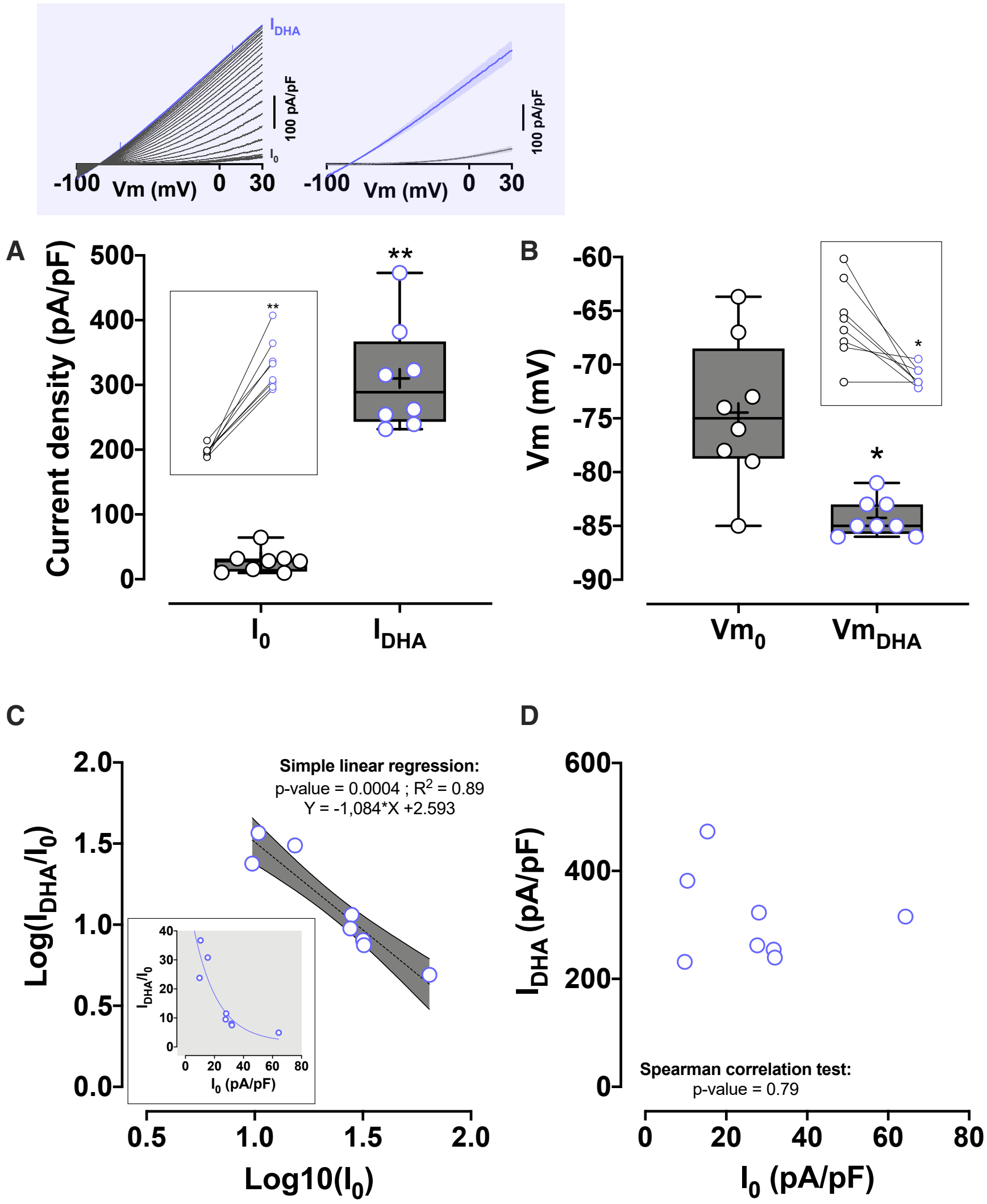

Figure 2. Characteristics of WT TREK-1 activation by DHA. (A) Current densities at 0 mV recorded in HEK-TREK-1_{WT} cells before (I_0, *dark circles*) and after (I_{DHA}, *purple circles*) perfusion with 10 μM DHA. The inset shows representative traces of the TREK-1 current during 5 min of activation by 10 μM DHA, with superimposed averages of I_0 and I_{DHA} after current stabilization. (B) Membrane potentials recorded in HEK-TREK-1_{WT} cells under basal conditions (Vm_0, *dark circles*) and following 10 μM DHA perfusion (Vm_{DHA}, *purple circles*). (A and B) Boxplots display the mean (*cross*), median (*horizontal line*), and individual data points (*circles*). Insets show before-and-after plots for individual cells. (C) Relationship between the magnitude of DHA-induced TREK-1 activation (I_{DHA}/I_0) and the initial current (I_0). Both parameters I_{DHA}/I_0 and I_0 are shown as log10-transformed values ($\log10(I_{DHA}/I_0)$ and $\log10(I_0)$). The relationship follows a Y = f(X) linear model, described by the regression equation $Y = -1.084 \times X + 2.593$. The dotted line represents the regression fit with 90% confidence intervals, and the associated *p*-value and R^2 are indicated. The inset shows a scatter plot representation of I_{DHA}/I_0 as a function of I_0 before log10 transformation. (D) Lack of correlation between the DHA-induced current density (I_{DHA}) at steady state and the initial current (I_0). The *p*-value for the Spearman correlation test is reported, whereas the correlation coefficient is not provided due to the nonsignificance of the test (*p*-value > 0.05).

initial current (I_0) and the ratio of the DHA-activated current (I_{DHA}/I_0) allows a straightforward assessment of potential power-law behavior. Moreover, because current amplitudes span a wide dynamic range, the log-log representation provides a more appropriate visualization and quantification of relative changes across conditions. In this representation, the observed linear trend indicates that the apparent hyperbolic decay seen on linear scales reflects an underlying scaling relationship. Simple linear regression statistically confirmed the relationship $\log10(I_{DHA,WT}/I_{0,WT}) = f(\log10(I_{0,WT}))$ in the transiently transfected cell model ($R^2 = 0.89$, *p*-value = 0.0004, Figure 2C). Moreover, the maximal DHA-activated current $I_{DHA,WT}$ appears independent of the initial current $I_{0,WT}$ (Spearman correlation test: *p*-value = 0.79, Figure 2D). Interestingly, DHA-induced activation reduces the variability of TREK-1 currents, with the CV decreasing from 63.9% for $I_{0,WT}$ to 26.8% for $I_{DHA,WT}$.

G171D TREK-1 mutation prevents TREK-1 activation by PUFAs

To investigate the mechanism underlying the variability in $I_{0,WT}$ and the PUFA-mediated effects, we used the TM2 G171D variant of TREK-1. This variant is characterized by a significant increase in *N*.Po at rest without affecting channel trafficking to the membrane.[27] For this variant, the increase in *N*.Po led to an increase in $I_{0,G171D}$ in the whole-cell configuration of the patch-clamp technique (Figure 3A; $I_{0,WT} = 27.4 \pm 17.5$ pA/pF vs. $I_{0,G171D} = 88.9 \pm 40.5$ pA/pF at 0 mV). Interestingly, the G171D mutation reduced variability in initial activity, with the CV decreasing from 63.9% for HEK-TREK-1_{WT} ($n = 8$) to 45.6% for HEK-TREK-1_{G171D} ($n = 10$). The G171D variant remains responsive to 10 μM DHA (Figure 3B), but the resulting increase in current amplitude is much smaller than in HEK-TREK-1_{WT} ($I_{DHA,G171D}/I_{0,G171D} = 1.6 \pm 0.5$ vs. $I_{DHA,WT}/I_{0,WT} = 16.6 \pm 12.1$ at

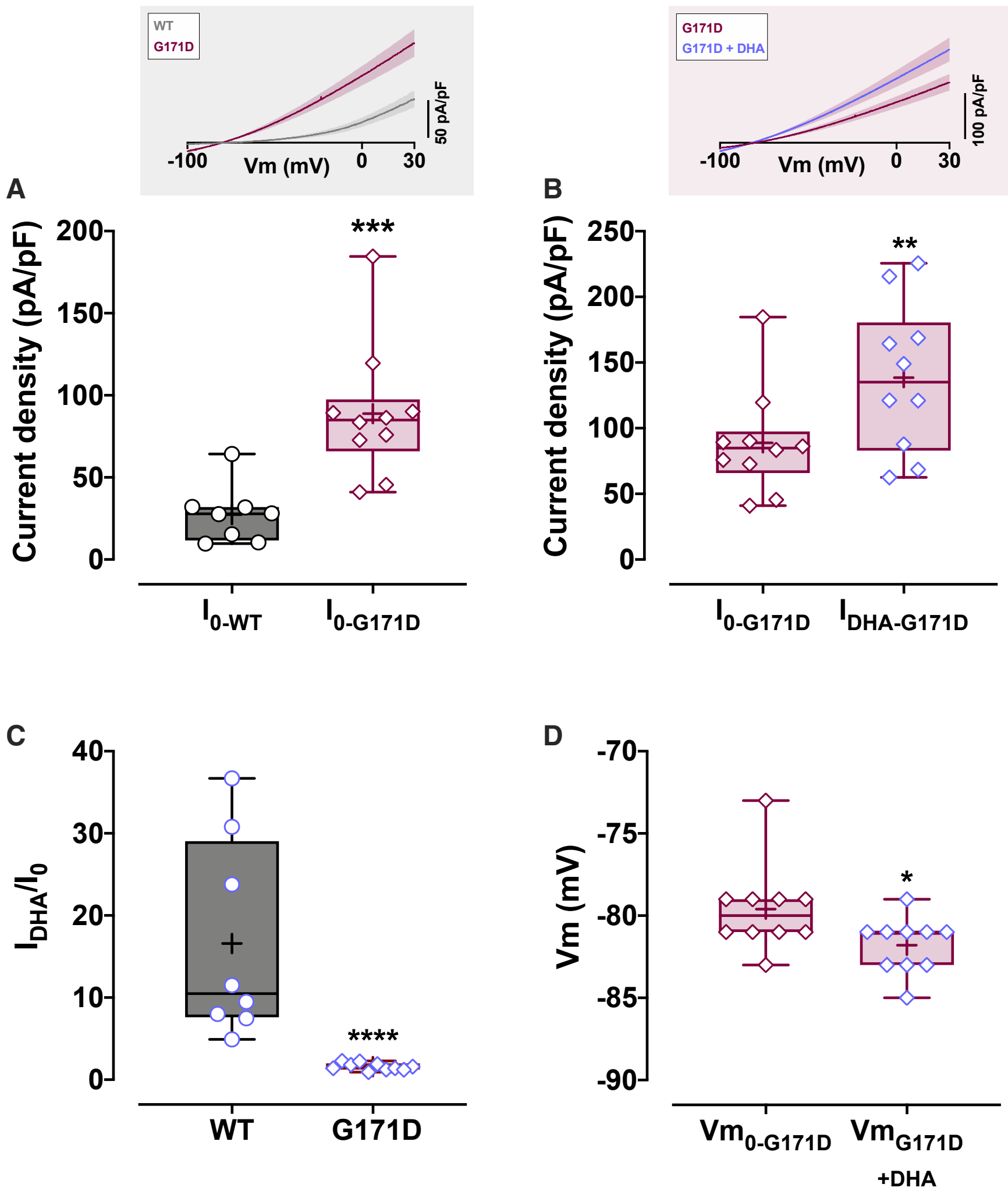

Figure 3. Comparison of DHA-induced activation of TREK-1 channels between HEK-TREK-1_{WT} and HEK-TREK-1_{G171D} cells. (A) Current densities at 0 mV under basal conditions recorded in HEK-TREK-1_{WT} ($I_{0,WT}$, *dark circles*) or HEK-TREK-1_{G171D} ($I_{0,G171D}$, $n = 10$, *red diamonds*). The inset shows the superposition of averages I_0 for HEK-TREK-1_{WT} ($n = 8$) and HEK-TREK-1_{G171D} ($n = 10$) cells (mean ± SEM). (B) Current densities at 0 mV recorded from HEK-TREK-1_{G171D} cells before ($I_{0,G171D}$, *red diamonds*) and after ($I_{DHA,G171D}$, *purple diamonds*) perfusion with 10 μM DHA. The inset shows the superposition of averages $I_{0,G171D}$ and $I_{DHA,G171D}$ (mean ± SEM, $n = 10$). (C) Magnitude of DHA-induced TREK-1 activation (I_{DHA}/I_0) in HEK-TREK-1_{WT} (*purple circles*, $n = 8$) and HEK-TREK-1_{G171D} (*purple diamonds*, $N = 10$) cells. (D) Membrane potentials recorded in HEK-TREK-1_{G171D} cells before (*red squares*) and after (*purple diamonds*) DHA perfusion ($n = 10$). (A–D) Boxplots display the mean (*cross*), median (*horizontal line*), and individual data points (*circles*).

0 mV; Figure 3C). As expected, the resting membrane potential of HEK-TREK-1_{G171D} cells is closer to the theoretical E_K than that of HEK-TREK-1_{WT} cells, with an average membrane potential $Vm_{0,G171} = -79.6 \pm 2.7$mV (Figure 3D; Data S1). Consequently, the smaller DHA-induced activation in HEK-TREK-1_{G171D} produces only a modest hyperpolarization ($Vm_{G171D} = -79.6 \pm 2.7$ mV vs. $Vm_{G171D\text{-}DHA} = -81.8 \pm 1.7$ mV) (Figure 3D). Therefore, these results indicate that the G171D mutation prevents strong DHA-mediated activation of TREK-1 channels.

Altogether, these data suggest a potential alteration of the negative relationship between $\log10(I_{DHA}/I_0)$ and $\log10(I_0)$ that we previously published.24 In HEK-TREK-1G171D, this negative relationship, typical of PUFA-induced activation in HEK-TREK-1_{WT}, is lost (Figure 4A). Conversely, regarding the G171D variant, the DHA-activated maximal current $I_{DHA,G171D}$ shows a positive linear correlation with the initial activity $I_{0,G171D}$ (Figure 4B).

Gating mode shift of TREK-1 induced by PUFAs

The TREK-1 current is characterized by outward rectification in the whole-cell configuration of the patch-clamp technique, resulting from a voltage-dependent gating mechanism.[3] As illustrated in Figure 5A, PUFA-mediated activation progressively abolishes the current rectification, leading to a linearization of the TREK-1 current-voltage relationship. Under control conditions, TREK-1 exhibits the characteristic outward rectification, associated with a low linearity coefficient of 0.1 ± 0.01 ($n = 8$). In contrast, DHA-induced activation significantly increases the linearity coefficient to 0.7 ± 0.1 ($n = 8$), indicating a conversion of TREK-1 gating from a voltage-dependent mode to an ohmic leak mode with a linear current-voltage relationship (Figure 5B). The current linearization develops progressively during TREK-1 activation and is fully reversible upon DHA washout (Figure 5C). In a previous study comparing the effects of multiple PUFAs on TREK-1 activity, we showed that DHA, together with the omega-6 fatty linoleic acid (LA), is among the strongest activators of TREK-1.[24] In

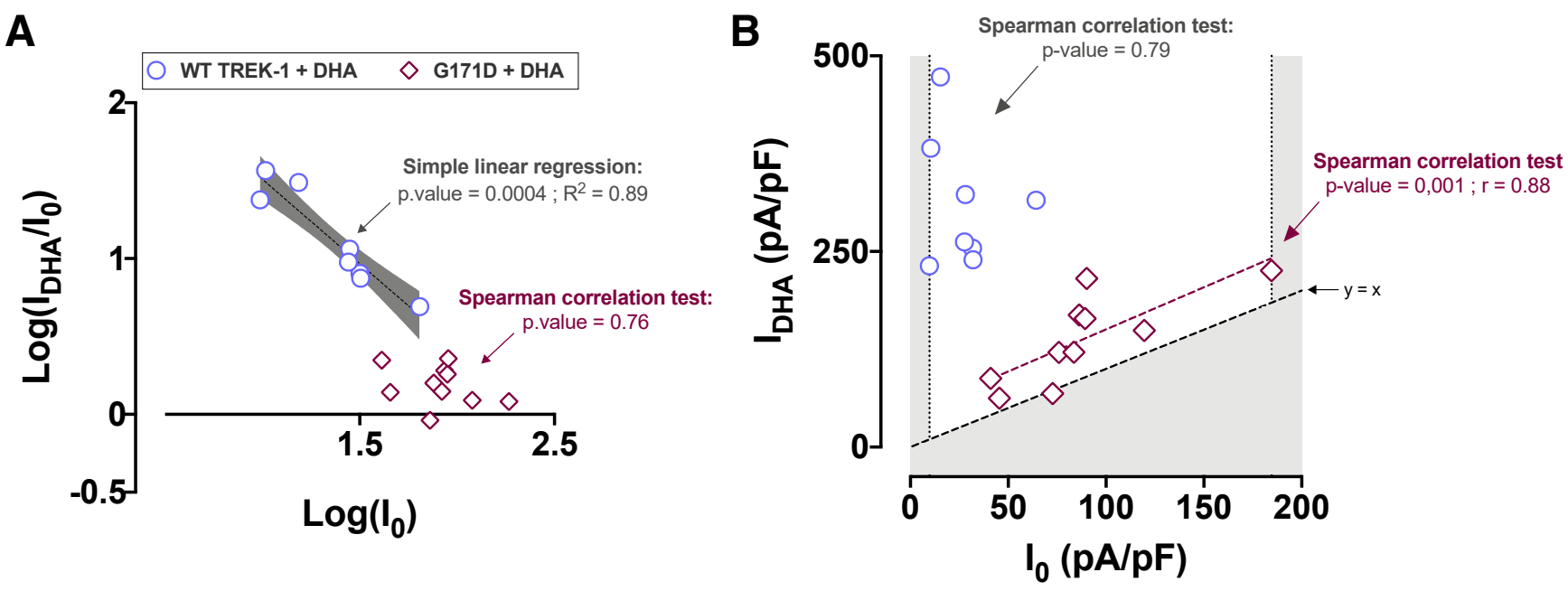

Figure 4. G171D mutation alters the activation of the TREK-1 channel by DHA. (A) HEK-TREK-1_{G171D} cells do not display (*red diamonds*) the negative relationship between the DHA-induced activation (I_{DHA}/I_0) and the initial current (I_0). The Spearman correlation test for the G171D variant was not significant (p-value $=$ 0.76), whereas WT TREK-1 (*purple circles*) follows a linear relationship described by a simple linear regression. (B) Correlation between the maximal current induced by DHA (I_{DHA}) and the initial current (I_0) in HEK-TREK-1_{WT} cells (*purple circles*) (p-value $=$ 0.001, n $=$ 8). In contrast, HEK-TREK-1_{G171D} cells (*red diamonds*) do not show a significant correlation (Spearman p-value $=$ 0.79, n $=$ 10).

contrast, weaker activators of TREK-1, such as eicosadienoic acid (EDA; C20:$2^{\Delta 6}$,[19]) and the synthetic compound ML402, do not induce linearization of the current-voltage relationship (Table S1). These findings indicate that only strong TREK-1 activators, such as DHA, are capable of reversibly converting TREK-1 gating to an ohmic leak mode (Figure 5C). Consistently, Figure 5D shows a progressive leftward shift of the TREK-1 activation curve toward hyperpolarized membrane potentials during DHA exposure. In control conditions, HEK-TREK-1_{G171D} cells already display reduced outward rectification ($I_{0,G171D}$), with a higher linearity coefficient of 0.4 $\pm$ 0.04 (Figure 6A, n $=$ 10). Although the G171D mutation does not abolish DHA sensitivity, the resulting activation remains weak ($I_{DHA,G171D}/I_{0,G171D}$ $=$ 1.6 $\pm$ 0.1; see Figure 3C) and is associated with only a modest increase in the linearity

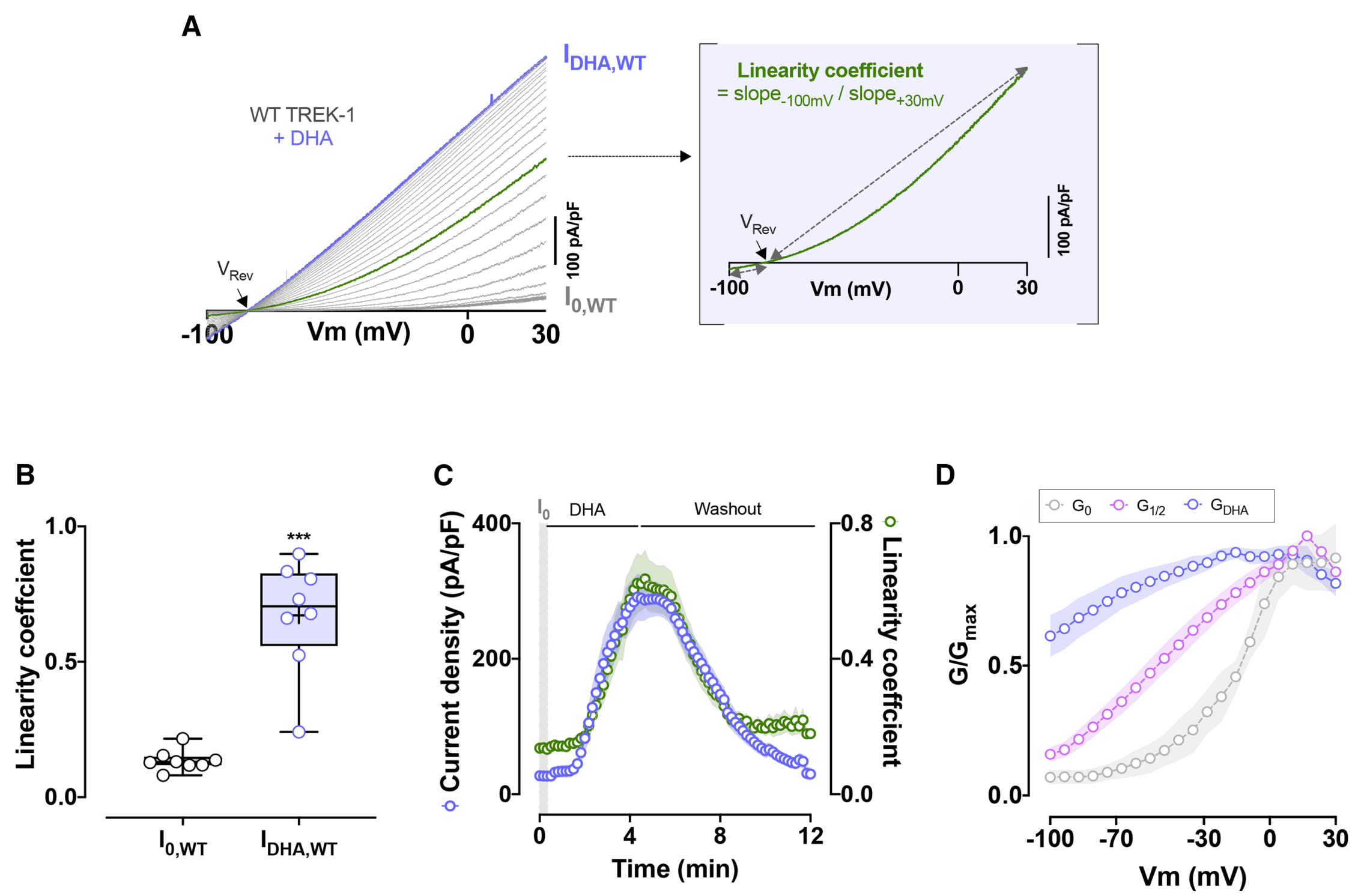

Figure 5. TREK-1 gating shifts from voltage-dependent to ohmic mode upon DHA activation. (A) Left: representative current traces recorded over 5 min from a HEK-TREK-1_{WT} cell during DHA activation. The initial current ($I_{0,WT}$, *dark gray and bold*), the current at half-activation (*green*), and the current at steady state of the activation ($I_{DHA,WT}$, *purple*) are highlighted. The $I_{0,WT}$-V curve displays outward rectification, whereas the $I_{max,WT}$-V curve is linear. Right: the linearity coefficient of the current between -100 and $+30$ mV was calculated as the slope ratio from V_{Rev}. (B) Coefficients of linearity calculated before ($I_{0,WT}$, *dark circles*) and after ($I_{max,WT}$, *purple circles*) DHA activation. Boxplots display the mean (*cross*), median (*horizontal line*), and individual data points (*circles*). (C) Time course of TREK-1 activation by DHA showing current densities (*green circles*) and corresponding linearity coefficients (*blue circles*) (mean $\pm$ SEM). (D) Activation curves of TREK-1 at different time points: resting condition (G_0), half-activation ($G_{1/2}$), and steady state (G_{PUFA}) (mean $\pm$ SEM).

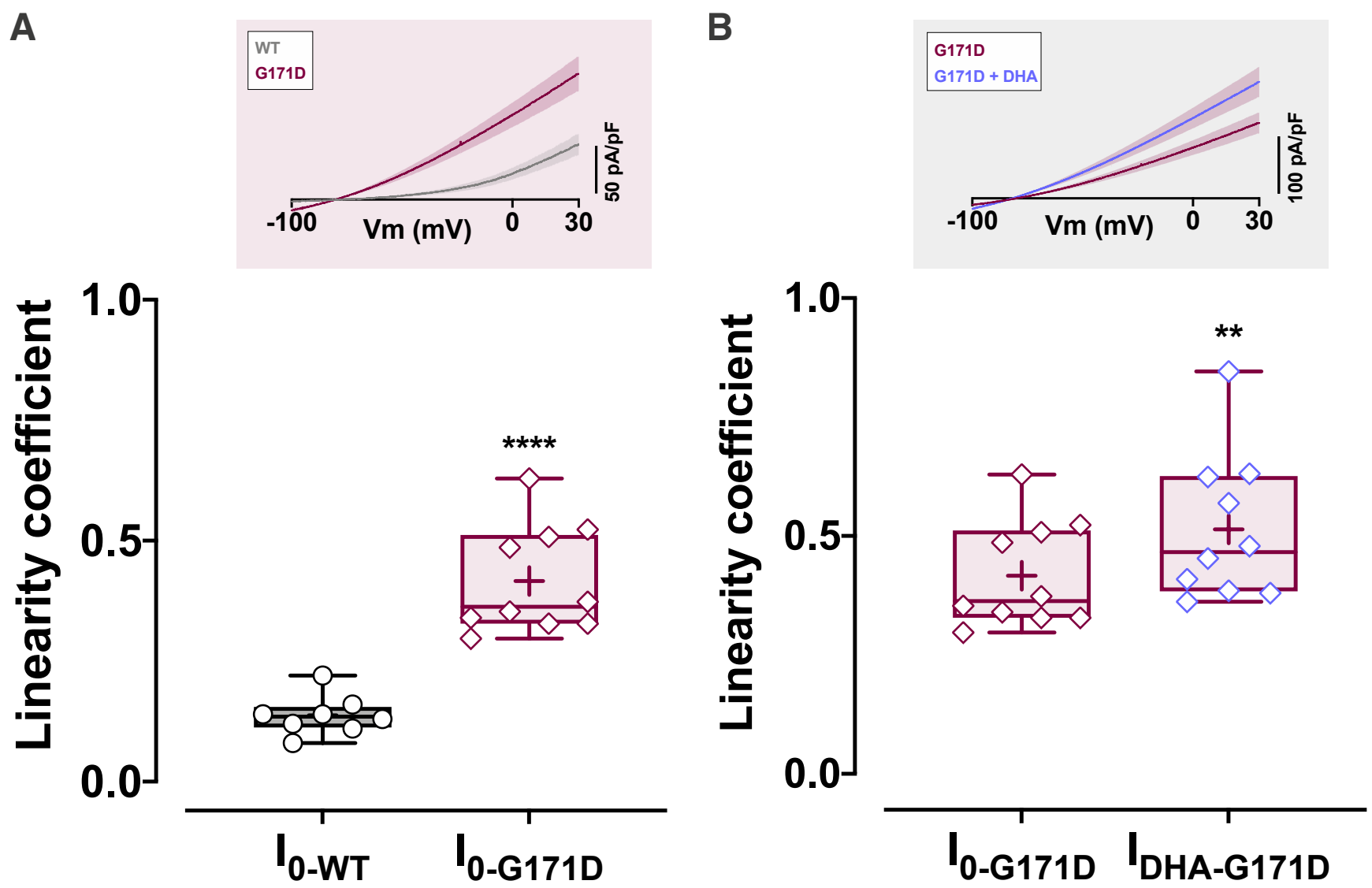

Figure 6. G171D mutation reduces the DHA-induced shift of TREK-1 from voltage-dependent to ohmic mode. (A) Linearity coefficients for the initial current recorded from HEK-TREK-1_{WT} ($I_{0,WT}$, *black circles*) or HEK-TREK-1_{G171D} ($I_{0,G171D}$, *red diamonds*). The inset shows the superposition of averages $I_{0,G171D}$ and $I_{0,WT}$ of TREK-1 (mean ± SEM, $n = 8$ and 10, respectively). (B) Linearity coefficients before ($I_{0,G171D}$, *red diamonds*) and after ($I_{DHA,G171D}$, *purple diamonds*) 10 μM DHA activation. The inset shows the superposition of averages $I_{0,G171D}$ and $I_{DHA,G171D}$ (mean ± SEM, $n = 10$). (A and B) Boxplots display the mean (*cross*), median (*horizontal line*), and individual data points (*circles* or *diamonds*).

coefficient (Figure 6B) compared with the WT channel (see Figure 5B). Together, these results suggest that DHA becomes less effective at inducing a full gating mode switch when the structure and function of the selectivity filter are altered, supporting a critical role of this region in PUFA-mediated modulation of TREK-1.

A conductance model to explain channel recruitment by PUFAs

To further elucidate the mechanism underlying variability in TREK-1 activation magnitude in response to PUFAs, we propose a conductance-based model derived from our experimental observations. Indeed, data obtained from HEK-TREK-1_{WT} cells indicate that the magnitude of PUFA-induced activation is inversely related to the initial activity of TREK-1: cells exhibiting lower initial activity display stronger activation upon PUFA perfusion. To account for this behavior, we developed a dynamic model of TREK-1 conductance in which PUFA activation is described as a progressive recruitment of channels into a conductive state, thereby increasing total conductance. In this framework, conductance is described as a time-dependent variable rather than a voltage-dependent one. Accordingly, we defined the total conductance G as

$$I_i = G_i \,.\, (V_m - E_k), \text{ where} \qquad (4)$$

$$G_i = \gamma_i \,.\, (N.Po)_i. \qquad (5)$$

Here, *I* represents the macroscopic current, γ is the unitary conductance, and *N*.Po defines the fraction of open channels. The index *i* denotes the simulated cell, with *i* ranging from 1 to 30.

We previously hypothesized that the variability in the initial activity of TREK-1 arises from cell-to-cell differences in the fraction of open channels, represented by the $(N.Po)_i$ term (Figure 1B). Accordingly, we propose that PUFAs activate TREK-1 by increasing the fraction of channels contributing to the total conductance, thus explaining the observed inverse relationship between $I_{DHA,WT}/I_{0,WT}$ and the initial current $I_{0,WT}$. Based on this hypothesis, we defined the simulation parameters G_0 and G_{max}, and each simulation was repeated 30 times ($n_{sim} = 30$):

$$G_{0,i} = \gamma \,.\, N_{tot} \,.\, P_{0,i} \text{ and} \qquad (6)$$

$$\overline{G_0} = \frac{1}{n} \,.\, \sum_{i=1}^{n} G_{0,i} = \overline{G_0} = \frac{1}{30} \,.\, \sum_{i=1}^{30} G_{0,i}. \qquad (7)$$

Assuming a constant unitary conductance γ from cell to cell, the average initial conductance $\overline{G_0}$ can be expressed as a function of the fraction of open channels ($N_{tot} \,.\, \overline{Po_0}$), where N_{tot} is the total number of channels distributed across the membrane and Po ϵ]0;1[is the open probability of individual channels[26,27] (Figure 7Aa). Then, assuming that γ remains constant from $G_{0,i}$ to $G_{max,i}$, the average conductance $\overline{G_{max}}$ at steady state during simulated activation is defined by two populations of open channels:

$$G_{max,i} = \gamma \,.\, (N_{PUFA} \,.\, Po_{max,i} + (N_{tot} - N_{PUFA}).Po_{0,i}) \text{ and} \qquad (8)$$

$$\overline{G_{max}} = \gamma \,.\, \left(\frac{1}{n} \sum_{i=1}^{n} N_{PUFA} \,.\, Po_{max,i} + \frac{1}{n} \sum_{i=1}^{n} (N_{tot} - N_{PUFA}) \,.\, Po_{0,i} \right), \text{ in which} \qquad (9)$$

$\overline{[N_{PUFA} . Po_{max}]}$ is the fraction of PUFA− activated channels,

where $Po_{max} > Po_0$ and $N_{PUFA} \leq N_{tot}$ and

$\overline{[(N_{tot} - N_{PUFA}) . Po_0]}$ is the fraction of non − activated

channels that maintain the initial open probability Po_0.

In both stable lines and HEK-TREK-1_{WT}, we observed that the initial TREK-1 current follows a beta distribution (Data S2A):

$$I_{0,WT} \sim B(\alpha, \beta), \text{with } \alpha < \beta.$$

Accordingly, we simulated $G_{0,i}$ values ($n_{sim} = 30$) with a high CV (>60%) to mirror the variability of $I_{0,WT}$ observed in HEK cells. The distribution of G_0 was modeled using a beta distribution with shape parameters $\alpha < \beta$, consistent with the positively skewed distribution of $I_{0,WT}$ (Figure 7Aa). The expected $\mathbb{E}(G_0)$ from this distribution is 10.02 ± 7.18 a.u. and corresponds to $\mathbb{E}(N_{tot}. Po_0)$. We then simulated two levels of activation, a strong and a weak effect, by pairing $G_{max,i}$ and $G_{0,i}$ values. The difference between strong and weak activation was modeled by varying the proportion of PUFA-activated channels:

$$N_{PUFA,strong} = 0.9 . N_{tot} \text{ and}$$

$$N_{PUFA,weak} = 0.6 . N_{tot}.$$

consistent with the progressive activation of TREK-1 by PUFAs (Figure 5C). The insets in Figure 7Ac illustrate the dynamics of channel activation at three time points: T_0, $T_{1/2}$, and $T_{steady\text{-}state}$. At T_0, all N_{tot} channels are present with a very low open probability ($\mathbb{E}(Po_0) = 0.020 \pm 0.014$), resulting in a low average conductance $\overline{G_0}$. The corresponding fraction of open channels, $\overline{N_{tot} . Po_0}$, is represented by yellow pixels in the left inset of Figure 7Ac. At $T_{1/2}$, the number of activated channels increases, denoted as $N_{PUFA1/2}$, leading to a rise in their open probability toward Po_{max}. This transition results in an increase in the average conductance $\overline{G_{1/2}}$ relative to the baseline $\overline{G_0}$. The fraction of activated and open channels, $\overline{[N_{PUFA} . Po_{max}]}_{1/2}$ at $T_{1/2}$, is depicted by purple pixels, whereas the fraction of nonactivated channels $\overline{[(N_{tot} - N_{PUFA}) . Po_0]}$ retains its initial open probability and remains shown in yellow (Figure 7Ac, *middle inset*). Finally, at $T_{steady\text{-}state}$, the majority of channels are activated and represented by the $\overline{[N_{PUFA} . Po_{max}]}$ term (*purple pixels*), while only a small fraction of nonactivated channels remains, shown in yellow (Figure 7Ac, *right inset*).

Figure 7B shows the lack of correlation between $G_{0,i}$ and $G_{max,i}$ as defined in our model, consistent with the lack of correlation observed experimentally between I_0 and I_{DHA} in HEK-TREK-1_{WT} cells (Figure 4B). In contrast, a nonlinear negative relationship is observed between $G_{max,i}/G_{0,i}$ and $G_{0,i}$, which is characteristic of PUFA-mediated TREK-1 activation (Figure 2C). This relationship follows a decay law of the form $\sim \frac{a}{x} + b$ (Figure 7C):

$$\frac{G_{max}}{G_0} = \frac{\gamma . ([N_{PUFA}. Po_{max}] + [(N_{tot} - N_{PUFA}). Po_0])}{\gamma . N_{tot} . Po_0} = \frac{([N_{PUFA}. Po_{max}] + [(N_{tot} - N_{PUFA}). Po_0])}{N_{tot} . Po_0}$$
$$= \frac{N_{PUFA}}{N_{tot}} . \frac{Po_{max}}{Po_0} + b, \text{where } b = \left(1 - \frac{N_{PUFA}}{N_{tot}}\right) = \frac{a}{G_0} + b, \text{where } a = N_{PUFA}.Po_{max}.$$

These two levels of activation were designed to account for the distinct effects of PUFAs described in our previous study.[24] Among the various PUFAs tested on TREK-1 channel activity, we identified at least two categories of activators: strong activators, such as DHA and LA (C18:2 n-6), and weak activators, such as EDA (C20:2 n-6) or ETA n-3 (eicosatetraenoic acid, C20:4 n-3). Figure 7Ac shows the simulated activation kinetics from $\overline{G_0}$ to $\overline{G_{max}}$, modeled as

$$\overline{G_{(t)}} = \gamma(\overline{[N_{PUFA} . Po_{max}]}_{(t)} + \overline{[(N_{tot} - N_{PUFA}) . Po_0]}_{(t)}). \quad (10)$$

This time course of activation is governed by a sigmoidal increase in N_{PUFA} over time (see materials and methods),

This negative relationship could explain why cells with low initial activity exhibit a greater fold activation in response to PUFA stimulation. The inverse relationship is linearized by a log10 transformation (Figure 7D):

$$\log 10\left(\frac{G_{max}}{G_0}\right) = f(\log 10(G_0)).$$

Figure 7E shows the linearized relationships between G_{max}/G_0 and G_0 for both strong and weak activation effects. The differences in the proportion of activated channels (N_{PUFA}) between strong and weak effects likely account for the observed differences in the slopes of these linear relationships, consistent with our previous observations on the effects of multiple PUFAs on TREK-1 activity.[24]

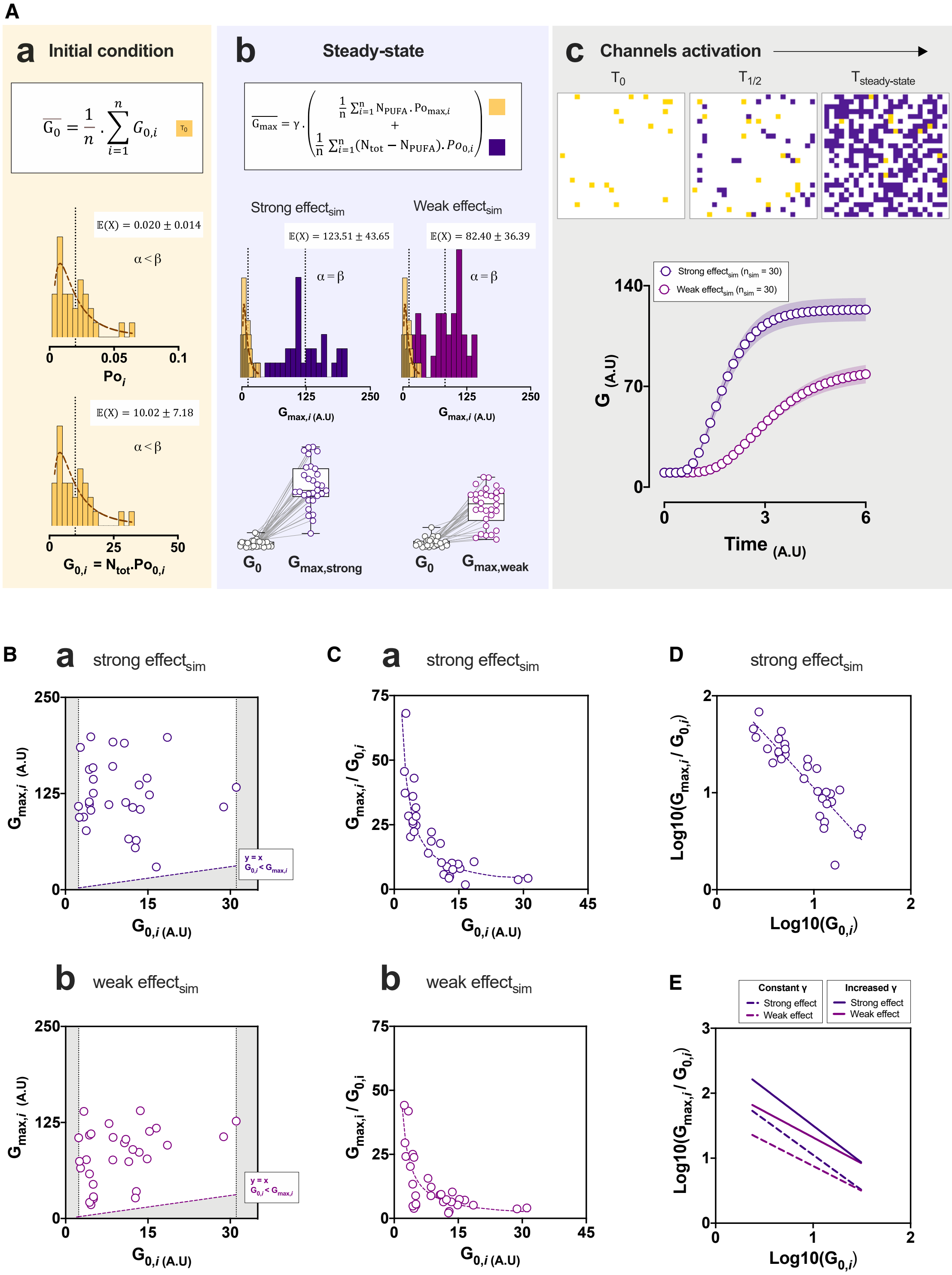

Figure 7. Mathematical model to explain TREK-1 channel recruitment by PUFAs. (Aa) Average $\overline{G_0}$ initial conductance is simulated using the equation $\overline{G_0} = \gamma\,(\overline{N_{tot}\,.\,Po_0})$. The distribution of individual initial conductance G_{0i} is determined by the distribution of Po_i, represented by yellow histograms (frequency distributions). Both Po_0 and G_0 follow a beta distribution with shape parameters $\alpha < \beta$, reflecting skewed distributions. (Ab) Simulated activation kinetics are shown for strong and weak simulated effects. The steady-state conductance $\overline{G_{max}}$ after activation is modeled as $\overline{G_{max}} = \gamma.([\overline{N_{PUFA}\,.\,Po_{max}}] + [(\overline{N_{tot} - N_{PUFA}})\,.\,Po_0])$, where $\overline{N_{PUFA}}$ represents the number of activated channels over time. The insets illustrate channel activation dynamics at three time points: T_0, yellow pixels represent initially open channels $(N_{tot}.\,\overline{Po})_0$; $T_{1/2}$ and $T_{steady\text{-}state}$, yellow pixels represent the $[(\overline{N_{tot} - N_{PUFA}})\,.\,Po_0]$ channels that maintain their initial open probability Po_0 but are not activated, while purple pixels indicate $[\overline{N_{PUFA}\,.\,Po_{max}}]$ activated channels. (Ac) Distributions of simulated G_{max} under strong and weak activation effects following symmetric beta distributions ($\alpha = \beta$). Boxplots illustrate the conductance increase from $\overline{G_0}$

(legend continued on next page)

To investigate the physiological relevance of TREK-1 current linearization by DHA, we simulated a cardiac action potential under 3 conditions (Figure 8). In this model, TREK-1 activation shortened the action potential duration and hyperpolarized the resting membrane potential, regardless of whether TREK-1 displayed outward rectification or a linear IV curve. However, these effects were stronger when the TREK-1 current was linearized.

DISCUSSION

Although K2P channels are constitutively open within the range of physiological membrane voltages, they exhibit complex gating properties. Often described as background or leak channels, voltage-dependent gating is common to most K2P channels.[3] However, unlike voltage-gated K^+ channels with 1P/6TM topology (Kv), K2P channels lack a canonical voltage sensor and do not undergo classical inactivation.[28] The principal gating mechanism of K2P channels is the selectivity filter gate, also known as C-type gating.[3,29,30] C-type gating involves conformational changes in the selectivity filter that influence ion conduction. In classical homotetrameric potassium channels, this mechanism is associated with conformational changes that lead to a nonconductive state. Structural studies have shown that C-type inactivation in classical potassium channels involves gradual conformational rearrangements in the selectivity filter backbone, ranging from filter pinching and dilation to more subtle structural shifts. These structural rearrangements disrupt key ion-binding sites, ultimately leading to a collapse of the conductive pathway and loss of potassium ion flow.[31,21] Conversely, K2P channels exhibit a unique gating mechanism due to the distinct selectivity filter architecture of the two pore-forming domains. In these channels, opening and closing of the selectivity filter gate arise from asymmetric, potassium-dependent order-disorder transitions within the selectivity filter, facilitated by movement of the M4 helix.[32–35] Moreover, the dynamic behavior of the selectivity filter is also critical for the modulation of channel activity by various stimuli. Synthetic modulators of TREK-1, such as ML402/ML335, act as a wedge at the P1-M4 interface, stabilizing the selectivity filter gate in an active permeating state and resulting in the ohmic mode of the channel.[30] Other natural modulators of TREK-1 similarly induce shifts in gating mode toward the ohmic mode, and vice versa. The negatively charged PIP_2 (phosphatidylinositol 4,5 bisphosphate) from the lipid bilayer interacts with positively charged residues of the C-terminal domain, shifting the voltage dependence of TREK-1 to more negative potentials.[22,36,37] Conversely, PKA-mediated phosphorylation on the S333 residue in the C-terminal domain converts TREK-1 from a leak-type gating mode to a voltage-dependent gating mode.[2] In this study, we describe that PUFA-induced activation produces a similar progressive and reversible shift in gating mode, converting TREK-1 from an ion-flux gated state to a more linear, ohmic conductance mode associated with a loss of the outward rectification. Specifically, upon activation by DHA, the TREK-1 activation curve progressively shifts toward more hyperpolarized membrane potentials, consistent with suppression of the ion-flux gated mechanism. The extent of this gating conversion is PUFA dependent: potent activators such as DHA and LA induce a stronger linearization of the current-voltage relationship and a greater shift in the activation curve, whereas weaker activators, such as ETA or the synthetic compound ML402, produce smaller effects.[24] These findings suggest that certain TREK-1 ligands are more effective at stabilizing the conductive state, either through distinct conformational rearrangements or via differences in binding affinity and coupling efficiency. We also report that the initial activity state of TREK-1 channels inversely affects the magnitude of PUFA-induced activation, following a law of decay of the form $\sim\frac{a}{G_0}+b$, where "a" represents the fraction of open channels at steady state and "b" corresponds to the proportion of nonactivated channels over N_{tot}. Our model suggests that, from cell to cell, a variable fraction of TREK-1 channels that do not contribute to the initial $I_{0,WT}$ becomes activated during DHA perfusion. Therefore, PUFAs act by increasing the number of channels in a conductive state at all voltages, but the proportion of activated channels is PUFA dependent, most likely reflecting differences in ligand affinity and the respective probabilities of interaction conferred by their three-dimensional structures.

Mutations in K2P channels have been reported to directly affect the C-type gate dynamics and modify channel activity by altering the coupling between the M4 helix and the selectivity filter.[34,35] Here, we report that a gain-of-function mutant of TREK-1 (TREK-1_{G171D}) at the TM2.6 position, near the intracellular part of the selectivity filter, partially suppresses the ion-flux gating mechanism, resulting in channel hyperactivation and a shift toward an ohmic, leak-like mode. The G171D variant of TREK-1, which exhibits constitutively increased open probability at the single-channel level, is significantly more active across all membrane potentials.[27] Interestingly, TREK-1_{G171D} displays ohmic behavior with a linear current-voltage relationship in the whole-cell configuration of patch-clamp experiments, highlighting a critical role of G171 in C-type gating. Moreover, this TM2.6 mutation alters PUFA-mediated gating by

to $\overline{G_{max}}$, with $G_{0,i} < G_{max,i}$. (B) Scatter plots (a and b) show the independence between $G_{max,i}$ and $G_{0,i}$ for both strong and weak activation effects. (C) Scatter plots of activation amplitude $G_{max,i}/G_{0,i}$ as a function of $G_{0,i}$. (D and E) Log-log linearized relationships between $G_{max,i}/G_{0,i}$ and $G_{0,i}$ after a log10 transformation, modeled as $\log10(G_{max,i}/G_{0,i}) = f(\log10(G_{0,i}))$. Strong and weak effects are shown, with constant or increased γ used to model $G_{max,i}$ from $G_{0,i}$.

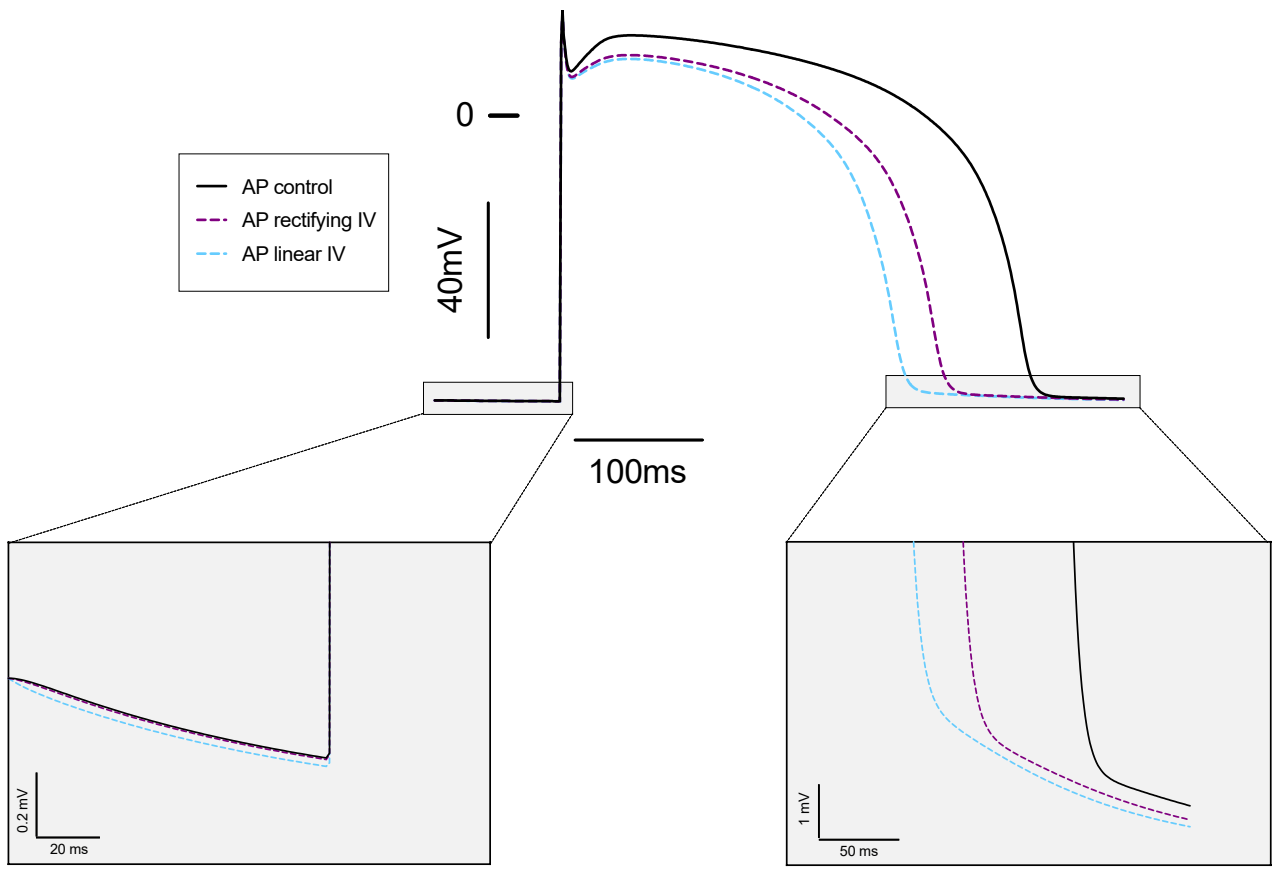

Figure 8. Model of TREK-1 current linearization effect on the ventricular action potential shape. Simulated ventricular action potentials (APs) under three conditions are shown: control without TREK-1 conductance (AP control, *black*), with TREK-1 conductance exhibiting outward rectification (AP curved IV, *pink*), and with TREK-1 conductance linearized across Vm (AP linear IV, *purple*).

reducing activation without completely abolishing it. Taken together, these results suggest that the G171D structure represents a conformation of a conductive channel and that PUFA-induced activation likely arises from stabilization of the C-type gate in a conductive state, potentially involving the TM2.

Studies on the voltage-dependent KCNQ1/KCNE1 channel indicate that PUFAs modulate channel activity in a manner analogous to their activation of the TREK-1 channel. Binding of the PUFA analog linoleoyl-glycine to KCNQ1 not only shifts the voltage dependence of activation[38] but also enhances the maximal conductance by promoting interactions that stabilize the open and conductive state of the selectivity filter.[23] This stabilization of the selectivity filter facilitates the transition from a nonconductive to a conductive state, thereby increasing channel open probability during PUFA perfusion. Complementing these functional observations, structural studies have shown that channels from both the K2P and Kv families possess equivalent regulatory sites capable of interacting with lipids to modulate channel gating. Recent work on K2P channels demonstrated that members, including THIK1 and TREK channels, share key architectural features with voltage-gated potassium channels such as KCNQ1, particularly in the pore region surrounding the selectivity filter, supporting mechanistic convergence in their gating behaviors.[39] A central unifying feature of these channels is the presence of a conserved interdomain ligand-binding site. In KCNQ1, PUFA-binding site II, which stabilizes the selectivity filter, corresponds to the P1-M4 PUFA site in THIK1 and to the synthetic modulator pocket described in TREK channels.[39]

Structurally, the TREK-1 channel exhibits multiple open states that can be induced by various stimuli.[34,37] Two major conformations are well established: a down state, in which the M2 and M4 helices are oriented downward, and an up state, characterized by an outward expansion toward the membrane, primarily involving M4. Despite these structural differences, the channel remains conductive in both conformations. In addition, intermediate states between the down-state and up-state conformations have been described.[37] The coexistence of multiple conductive states indicates that channel activation does not follow a simple open/closed mechanism but rather reflects a shift in the equilibrium between these states. Accordingly, under basal conditions, the channel already exists in a partially conductive equilibrium, with a nonzero but low open probability. DHA shifts this equilibrium toward more conductive states, thereby increasing the open probability. As captured by our model, DHA-induced activation results in a redistribution of the channel's opening probability across its available states. This redistribution accounts for the inverse relationship between activation amplitude and initial activity. The observed decay law relating I_{max}/I_0 to I_0, which becomes linear upon logarithmic transformation, arises from the DHA-induced changes in opening probability. Thus, DHA should not be viewed as a simple activator but rather as a state-dependent modulator that acts according to the channel's initial condition. In this sense, it functions as a homeostatic regulator, enhancing weak activity while limiting strong activity, thereby shaping the dynamic range of TREK-1 channel function.

Ultimately, the findings of this work suggest that TREK-1 activation by PUFAs under physiopathological conditions can have several outcomes depending on the channel's initial activity. Under physiological conditions, voltage-dependent potassium channels contribute to the repolarization of the membrane potential (Vm), while leak channels maintain Vm closer to E_K, thereby keeping it further from the threshold for action potential initiation. The outward rectification of TREK-1 arises from its voltage dependence at depolarized potentials, allowing it to contribute not only to the establishment of the resting membrane potential (Vm) but also to the repolarization phase of the action potential.[40] Regarding PUFA-induced gating, the interconversion of TREK-1 between voltage-dependent and ohmic modes provides a mechanism for dynamic regulation of cellular excitability. Through this mechanism, PUFAs may contribute to the suppression of excitatory responses. Using a model of ventricular action potential, we observed that the ohmic mode of TREK-1 can further shorten action potential duration. Figure 8 illustrates how TREK-1 conductance in leak versus voltage-dependent modes influences the ventricular action potential. When the channel adopts an ohmic, leak-like mode at the same conductance, the action potential is shortened. This shortening may have dual effects: it may be pro-arrhythmic by reducing the refractory period or anti-arrhythmic by preventing the occurrence of early afterdepolarizations.

Finally, this model supports the hypothesis that (1) variability in the fraction of open channels at rest underlies the observed distribution of I_0 and (2) PUFAs increase the TREK-1 current by acting through an $N_{PUFA}.Po_{max}$ mechanism, resulting in the variable PUFA-mediated activation of TREK-1. According to this model, the difference between strong activation by DHA and weaker activation by other PUFAs can be explained by a higher $N_{PUFA}.Po_{max}$ induced by DHA (Figure 7).

DATA AVAILABILITY

Data supporting the model presented in Figure 7 have been deposited in a publicly accessible repository and are available at https://github.com/EmilieDrim/2025_Model-of-TREK-1-activation-by-PUFAs. All other data analyzed during this study are available from the corresponding author upon reasonable request.

ACKNOWLEDGMENTS

This work was supported by the Agence Nationale de la Recherche under the project acronym TA-BOOm (ANR-20-CE44-0001) and by the CBS2 Doctoral School. We thank Nicolas Paillet, research engineer at the Néel Institute, for his valuable assistance and insightful guidance in the modeling work (Figure 7). We are grateful to Prof. Okada for the generous gift TR-1 cells, a HEK293T cell line stably expressing the TREK-1 channel (Data S2). We also warmly thank Thomas Boulin, who first described the G171D mutation of the TREK-1 channel and suggested comparing WT and mutant responses to PUFAs. The graphical asbtract was created with BioRender.com.

AUTHOR CONTRIBUTIONS

Performed experiments, E.B.; designed research, E.B., J.-Y.L.G., and M.D.; performed models, E.B. and J.-Y.L.G.; contributed to analytic tools, E.B., J.-Y.L.G., and M.D.; analyzed data, E.B. and J.-Y.L.G.; wrote the manuscript, E.B., J.-Y.L.G., and M.D.; supervised project and acquired funding, J.-Y.L.G. and MD.

DECLARATION OF INTERESTS

The authors declare no competing interests.

SUPPLEMENTAL INFORMATION

Supporting material can be found online at https://doi.org/10.1016/j.bpj.2026.05.005.

REFERENCES

1. Lesage, F., and M. Lazdunski. 2000. Molecular and functional properties of two-pore-domain potassium channels. *Am. J. Physiol. Renal Physiol.* 279:F793–F801.
2. Bockenhauer, D., N. Zilberberg, and S. A. Goldstein. 2001. KCNK2: reversible conversion of a hippocampal potassium leak into a voltage-dependent channel. *Nat. Neurosci.* 4:486–491.
3. Schewe, M., E. Nematian-Ardestani, …, T. Baukrowitz. 2016. A Non-canonical Voltage-Sensing Mechanism Controls Gating in K2P K+ Channels. *Cell.* 164:937–949.
4. Heurteaux, C., N. Guy, …, M. Lazdunski. 2004. TREK-1, a K+ channel involved in neuroprotection and general anesthesia. *EMBO J.* 23:2684–2695.
5. Marsh, B., C. Acosta, …, S. N. Lawson. 2012. Leak K^+ channel mRNAs in dorsal root ganglia: relation to inflammation and spontaneous pain behaviour. *Mol. Cell. Neurosci.* 49:375–386.
6. Herrera-Pérez, S., A. Campos-Ríos, …, J. A. Lamas. 2021. Contribution of K2P Potassium Channels to Cardiac Physiology and Pathophysiology. *Int. J. Mol. Sci.* 22:6635.
7. Khoubza, L., F. C. Chatelain, …, D. Bichet. 2021. Physiological roles of heteromerization: focus on the two-pore domain potassium channels. *J. Physiol.* 599:1041–1055.
8. Luo, Y., L. Huang, …, R. Jiang. 2021. Contribution of Neuronal and Glial Two-Pore-Domain Potassium Channels in Health and Neurological Disorders. *Neural Plast.* 2021:8643129.
9. Patel, A. J., E. Honoré, …, M. Lazdunski. 1998. A mammalian two pore domain mechano-gated S-like K+ channel. *EMBO J.* 17:4283–4290.
10. Bang, H., Y. Kim, and D. Kim. 2000. TREK-2, a New Member of the Mechanosensitive Tandem-pore K+ Channel Family. *J. Biol. Chem.* 275:17412–17419.
11. Maingret, F., I. Lauritzen, …, E. Honoré. 2000. TREK-1 is a heat-activated background K+ channel. *EMBO J.* 19:2483–2491.
12. Kang, D., C. Choe, and D. Kim. 2005. Thermosensitivity of the two-pore domain K+ channels TREK-2 and TRAAK. *J. Physiol.* 564:103–116.
13. Lengyel, M., G. Czirják, and P. Enyedi. 2020. TRESK and TREK-2 two-pore-domain potassium channel subunits form functional heterodimers in primary somatosensory neurons. *J. Biol. Chem.* 295:12408–12425.
14. Busserolles, J., I. Ben Soussia, …, S. Lolignier. 2020. TREK1 channel activation as a new analgesic strategy devoid of opioid adverse effects. *Br. J. Pharmacol.* 177:4782–4795.
15. Huang, L., G. Xu, …, J. Liu. 2022. Development of Non-opioid Analgesics Targeting Two-pore Domain Potassium Channels. *Curr. Neuropharmacol.* 20:16–26.
16. Alloui, A., K. Zimmermann, …, M. Lazdunski. 2006. TREK-1, a K+ channel involved in polymodal pain perception. *EMBO J.* 25:2368–2376.
17. Kamatham, S., C. M. Waters, …, S. Mancarella. 2019. TREK-1 protects the heart against ischemia-reperfusion-induced injury and from adverse remodeling after myocardial infarction. *Pflugers Arch.* 471:1263–1272.
18. Bechard, E., J. Bride, and M. Demion. 2022. TREK-1 in the heart: Potential physiological and pathophysiological roles. *Front Physiol.* 13:1095102.
19. Goonetilleke, L., and J. Quayle. 2012. TREK-1 K(+) channels in the cardiovascular system: their significance and potential as a therapeutic target. *Cardiovasc. Ther.* 30:e23–e29.
20. Lu, L., G. Zhang, and S. Zhou. 2017. Arachidonic acid has protective effects on oxygen-glucose deprived astrocytes mediated through enhancement of potassium channel TREK-1 activity. *Neurosci. Lett.* 636:241–247.
21. Hoshi, T., and C. M. Armstrong. 2013. C-type inactivation of voltage-gated K+ channels: pore constriction or dilation? *J. Gen. Physiol.* 141:151–160.
22. Chemin, J., A. J. Patel, …, E. Honoré. 2005. A phospholipid sensor controls mechanogating of the K+ channel TREK-1. *EMBO J.* 24:44–53.
23. Golluscio, A., J. Eldstrom, …, H. P. Larsson. 2024. PUFA stabilizes a conductive state of the selectivity filter in IKs channels. *eLife.* 13:RP95852.
24. Bechard, E., E. Arel, …, M. Demion. 2024. Activation of hTREK-1 by polyunsaturated fatty acids involves direct interaction. *Sci. Rep.* 14:15244.

25. Ten Tusscher, K. H. W. J., D. Noble, …, A. V. Panfilov. 2004. A model for human ventricular tissue. *Am. J. Physiol. Heart Circ. Physiol.* 286:H1573–H1589.

26. Xian, T. L., V. Dyachenko, …, J. Daut. 2006. The stretch-activated potassium channel TREK-1 in rat cardiac ventricular muscle. *Cardiovasc. Res.* 69:86–97.

27. Ben Soussia, I., S. El Mouridi, …, T. Boulin. 2019. Mutation of a single residue promotes gating of vertebrate and invertebrate two-pore domain potassium channels. *Nat. Commun.* 10:787. https://doi.org/10.1038/s41467-019-08710-3.

28. Snyders, D. J. 1999. Structure and function of cardiac potassium channels. *Cardiovasc. Res.* 42:377–390.

29. Piechotta, P. L., M. Rapedius, …, T. Baukrowitz. 2011. The pore structure and gating mechanism of K2P channels. *EMBO J.* 30:3607–3619.

30. Lolicato, M., C. Arrigoni, …, D. L. Minor. 2017. K2P2.1 (TREK-1)-activator complexes reveal a cryptic selectivity filter binding site. *Nature.* 547:364–368.

31. Cuello, L. G., V. Jogini, …, E. Perozo. 2010. Structural mechanism of C-type inactivation in K(+) channels. *Nature.* 466:203–208.

32. Bagriantsev, S. N., R. Peyronnet, …, D. L. Minor. 2011. Multiple modalities converge on a common gate to control K2P channel function. *EMBO J.* 30:3594–3606.

33. Bagriantsev, S. N., K. A. Clark, and D. L. Minor. 2012. Metabolic and thermal stimuli control K2P2.1 (TREK-1) through modular sensory and gating domains. *EMBO J.* 31:3297–3308.

34. Zhang, Q., J. Fu, …, H. Yang. 2022. 'C-type' closed state and gating mechanisms of K2P channels revealed by conformational changes of the TREK-1 channel. *J. Mol. Cell Biol.* 14:mjac002.

35. Lolicato, M., A. M. Natale, …, D. L. Minor. 2020. K2P channel C-type gating involves asymmetric selectivity filter order-disorder transitions. *Sci. Adv.* 6:eabc9174.

36. Lopes, C. M. B., T. Rohács, …, D. E. Logothetis. 2005. PIP2 hydrolysis underlies agonist-induced inhibition and regulates voltage gating of two-pore domain K+ channels. *J. Physiol.* 564:117–129.

37. Panasawatwong, A., T. Pipatpolkai, and S. J. Tucker. 2022. Transition between conformational states of the TREK-1 K2P channel promoted by interaction with PIP2. *Biophys. J.* 121:2380–2388.

38. Bohannon, B. M., X. Wu, …, H. P. Larsson. 2020. Polyunsaturated fatty acids produce a range of activators for heterogeneous IKs channel dysfunction. *J. Gen. Physiol.* 152:e201912396.

39. Roy-Chowdhury, S., S. Jang, …, D. L. Minor. 2025. Structure of the human K2P13.1 channel reveals a hydrophilic pore restriction and lipid cofactor site. *Nat. Struct. Mol. Biol.* 32:1154–1166.

40. MacKenzie, G., N. P. Franks, and S. G. Brickley. 2015. Two-pore domain po- tassium channels enable action potential generation in the absence of voltage- gated potassium channels. *Pflugers Arch.* 467:989–999.

Biophysical Journal, Volume *125*

Supplemental information

Gating shift to an ohmic mode of TREK-1 channel induced by docosa-hexaenoic acid

Emilie Bechard, Jean-Yves Le Guennec, and Marie Demion

SUPPLEMENTARY DATA

Supplementary Information 1: Th python script used to generate **Figure 7** is open-source (MIT license), hosted on GitHub, and available at the following link: https://github.com/EmilieDrim/2025_Model-of-TREK-1-activation-by-PUFAs

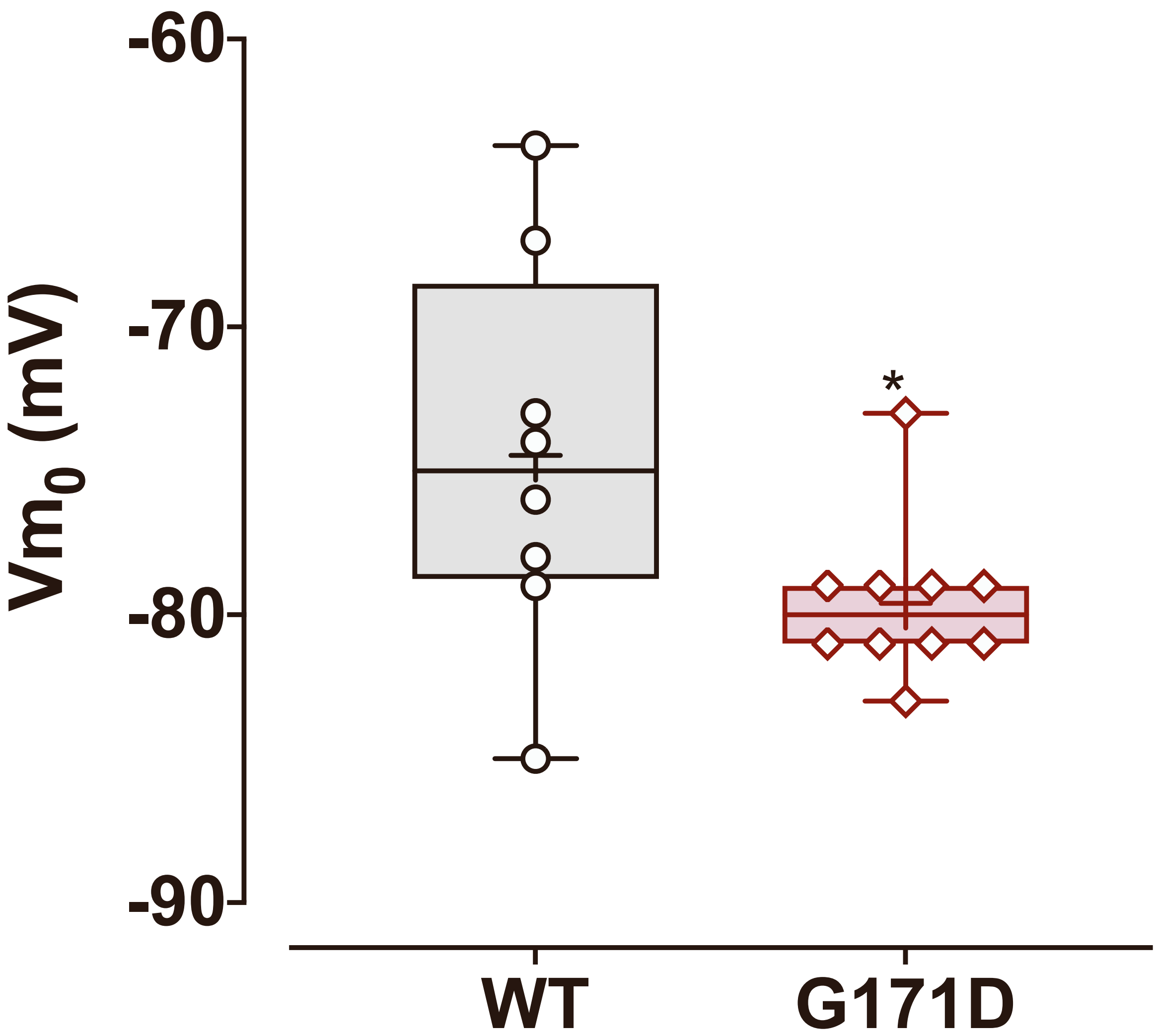

Supplemental data 1: G171D mutation hyperactivate TREK-1 channel
Resting membrane potentials of HEK293T cells transfected with WT TREK-1 (dark circles) or G171D TREK-1 (red diamonds) in resting conditions. Box plots display the mean (cross), median (horizontal line) and individual data points (circles).

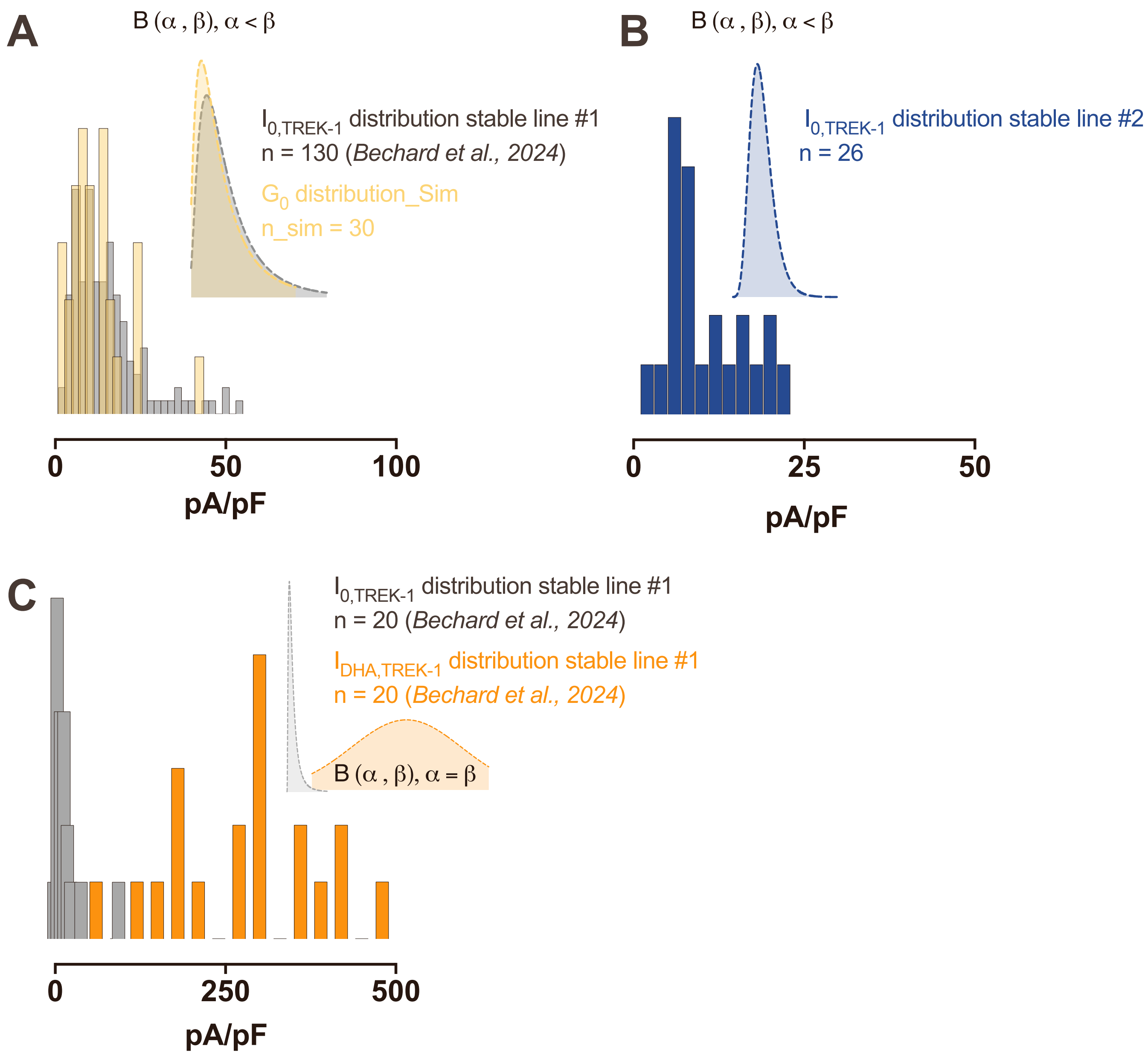

Supplemental data 2: Distributions of TREK-1 activity in HEK cell models

A. Distribution of TREK-1 current ($I_{0,TREK-1}$) in control conditions recorded at 0 mV from a stable HEK293 #1 line overexpressing TREK-1 channel (gray boxes, data from Bechard et al., 2024). Yellow boxes represent the distribution of modeled G_0 in Figure 7. **B.** Distribution of TREK-1 current ($I_{0,TREK-1}$) in control conditions recorded at 0 mV from a second HEK293 #2 stable line overexpressing TREK-1 channel (blue boxes). **C.** Superposition of $I_{0,TREK-1}$ (gray boxes) and $I_{DHA,TREK-1}$ (orange boxes) distributions, recorded from the HEK293 #1 stable line overexpressing TREK-1 (Bechard et al., 2024).

	**Cellular model**	**Reference**	**$I_{max}/I_{0\text{-}WT}$**	**Linearity coefficient of $I_{0,WT}$**	**Linearity coefficient of $I_{DHA,WT}$**
**DHA**	Transient transfection	Present study	16.58 ± 4.28	0.14 ± 0.01	0.67 ± 0.07
**DHA**	Stable HEK TREK-1 line	Bechard et al., 2024	29.79 ± 4.37	0.13 ± 0.009	0.58 ± 0.05
**LA**			24.76 ± 3.33	0.12 ± 0.008	0.53 ± 0.03
**ETA**			15.66 ± 18.78	0.12 ± 0.04	0.17 ± 0.07
**ML402**			9.56 ± 0.97	0.10 ± 0.01	0.21 ± 0.02

Supplemental Table 1: Linearization of TREK-1 current during activation by PUFAs and ML402.
